# Investigating feature-engineered predictors for systolic blood pressure changes in an mHealth-based disease management program

**DOI:** 10.1038/s41440-026-02569-w

**Published:** 2026-02-17

**Authors:** Masashi Kanai, Sangjun Park, Takahiro Miki, Yuta Hagiwara, Atsushi Hashimoto, Hidetaka Nambo, Shigehiro Karashima

**Affiliations:** 1 Institute of Transdisciplinary Sciences for Innovation, Kanazawa University, Kanazawa, Japan; 2 PREVENT Inc, Aichi, Japan; 3 Division of Electrical, Information and Communication Engineering, Graduate School of Natural Science and Technology, Kanazawa University, Kanazawa, Japan; 4 Institute of Liberal Arts and Science, Kanazawa University, Kanazawa, Japan

**Keywords:** mHealth, blood pressure change, prediction, machine learning, digital biomarker, morning hypertension, implemental hypertension, digital hypertension

## Abstract

Mobile health (mHealth)-based disease management programs enable continuous monitoring of blood pressure (BP) and related health behaviors. Feature engineering may help to extract informative predictors from longitudinal data, potentially improving BP change prediction. This study aimed to evaluate whether feature-engineered predictors can improve the prediction of systolic BP (SBP) changes using an mHealth-based disease management program. We analyzed data from participants with hypertension, dyslipidemia, or diabetes mellitus who completed the 24-week Mystar program, which combined phone-based coaching, remote monitoring, and app-based logging of BP and behavioral data. The primary outcome was the change in morning SBP from baseline to the end of the program. Prediction models for SBP changes were developed using ElasticNet regression at weeks 4, 8, 12, and 22 by comparing models with and without feature-engineered variables generated by feature tools. In total, 2318 participants were included in the analysis. At week 4, the top feature after feature engineering showed a stronger correlation with SBP change (r = 0.561) than the best original predictor (r = 0.455), although the model-level performance was similar (r = 0.561 vs. 0.559). By week 22, both models achieved a high correlation of approximately 0.85 with no substantial difference in performance. Feature engineering increased the correlation between individual predictors and SBP change in the early phase; however, the overall prediction performance of the ElasticNet model remained largely unchanged. Further studies are required to confirm these findings and examine their applicability in broader clinical and implementation contexts.

**Figure:**
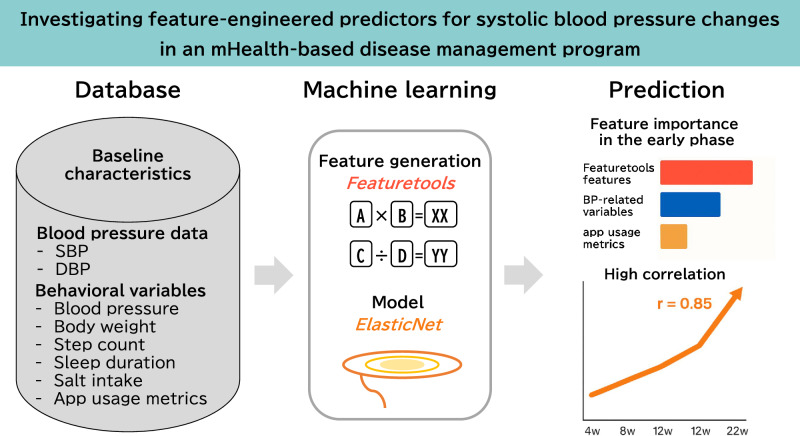
Data from a 24-week mHealth-based program were analyzed to predict systolic blood pressure SBP changes using feature-engineered variables and ElasticNet regression. In the early phase, feature-engineered predictors ranked highest in importance, although overall model performance remained similar with and without feature engineering. Prediction accuracy improved over time, with correlations reaching ~0.85 by week 22

Data from a 24-week mHealth-based program were analyzed to predict systolic blood pressure SBP changes using feature-engineered variables and ElasticNet regression. In the early phase, feature-engineered predictors ranked highest in importance, although overall model performance remained similar with and without feature engineering. Prediction accuracy improved over time, with correlations reaching ~0.85 by week 22

## Abstract (web summary)

These authors contributed equally: Masashi Kanai and Sangjun Park.

## Introduction

Hypertension is a leading risk factor for cardiovascular and cerebrovascular diseases [1–3] and a major contributor to the global burden [4, 5]. Despite advancements in treatment, the management of hypertension remains challenging owing to its multifactorial nature, involving genetic, environmental, and behavioral influences [6]. Conventional hypertension management primarily relies on periodic clinical visits and self-monitoring of blood pressure (BP) using home devices [7]. However, these approaches may not fully capture the dynamic nature of BP fluctuations, potentially resulting in suboptimal intervention strategies.

The emergence of mobile health (mHealth)-based disease management programs has facilitated the real-world continuous monitoring of BP and related health behaviors [8, 9]. These programs use mobile applications, wearable devices, and remote coaching to support lifestyle modifications. Integrating digital health technologies enables the collection of longitudinal BP data, offers new opportunities to analyze BP dynamics, and develops predictive models for BP changes [10, 11]. Identifying reliable predictors of BP changes within these programs could improve risk stratification and intervention strategies and ultimately enhance hypertension management.

Feature-engineering techniques can potentially reveal novel predictors of complex health data [12]. In clinical modeling, feature engineering refers to transforming raw signals into informative inputs to make underlying patterns more learnable for statistical or machine learning (ML) models. In particular, automated feature generation methods, such as those used in time-series analysis, may help systematically extract informative variables from BP data and other health-related factors. Recent studies have highlighted the utility of ML in modeling BP variability [10, 13]. However, the combined use of automated feature engineering and ML for BP change prediction remains underexplored. These methods may identify novel interpretable markers that serve as potential digital biomarkers for blood pressure management [14]. Such digital biomarkers enable remote, continuous monitoring and timely intervention rather than episodic care. These markers can provide insights into BP regulation and intervention efficacy. These markers provide insights into BP regulation and intervention efficacy. However, the extent to which feature-engineered variables improve BP prediction remains unclear.

This study focused on systolic blood pressure (SBP) as the primary outcome because it tends to exhibit greater variability and is more strongly associated with cardiovascular risk than diastolic blood pressure (DBP) [15, 16]. This study aimed to evaluate whether feature-engineered predictors could improve the prediction of SBP changes in an mHealth-based disease-management program. Additionally, we investigated whether the predictive advantage of feature-engineered predictors persisted throughout the intervention period or whether recent BP trends became a stronger determinant over time. Understanding the evolving predictive contributions of these variables may offer practical insights into optimizing data-driven approaches and refining personalized strategies for hypertension management.

Point of View

- Clinical relevance
  Recent home BP is the main signal, and engagement logs add a small extra clue to help decide who may need closer follow-up in mHealth programs when clinic visits are limited.
- Future direction
  Validate the model in other populations, and test whether risk-based support can be implemented in routine care with acceptable cost and workload.
- Consideration for the Asian population
  This approach may fit insurer- and workplace-based mHealth programs common in Asia, where morning hypertension and keeping participants engaged can be challenging.

## Methods

### Study design and participants

This study employed a retrospective cohort, pre-post observational design using data from PREVENT, Inc. (Nagoya, Japan), which provides medical data analyses and an mHealth-based disease management program for chronic conditions. PREVENT, Inc. predicted the risk of cerebrovascular and cardiovascular disease onset using health insurance claims and health checkup data. Employees or their dependents enrolled in health insurance associations participating in the lifestyle modification support program, called “Mystar,” run by PREVENT Inc., between December 2018 and November 2023, were screened for inclusion in the present study. The Mystar program targets individuals with hypertension, dyslipidemia, and diabetes mellitus who are taking medication or have a history of coronary artery disease or stroke [9]. In this study, we included participants who completed the Mystar program. We included participants who completed the Mystar program. Participants were excluded if [1] they had missing blood pressure measurements at the start or end of the program or [2] their weekly BP measurement frequency did not meet the required threshold for analysis.

The Institute of Transdisciplinary Sciences Ethics Committee of Kanazawa University approved this study (approval number: R6-003). The participants agreed to a privacy policy at the start of the program, which stated that the data gathered in the app may be used for future research.

### Details of the Mystar program

The Mystar program was implemented with the approval of the attending physicians. This program was provided over a six-month period through 12 phone call sessions with healthcare professionals held every two weeks, along with chat messaging between the sessions. Each participant was issued an account for a mobile app through which they logged lifestyle data, such as body weight, blood pressure, physical activity, salt intake, and food photographs. Healthcare professionals analyzed these data to identify key areas for lifestyle modification based on each participant’s situation, fostering communication that drives behavioral changes.

Within the app, participants could log their lifestyle data, monitor changes in their habits, review corrective goals and behavior-to-do lists, chat with their healthcare provider, and access disease management information provided by their coaches. The program targeted improvements in exercise, diet, sleep, alcohol consumption, smoking, and stress management, with personalized health plans developed for each participant. Further details have been provided in a previous study [9, 17].

### Outcome measurements

The primary outcome of this study was the change in SBP after the program. Participants were instructed by healthcare professionals on how to measure morning BP according to established guidelines [18]. They were asked to record their daily morning SBP and diastolic DBP using their own devices and input the values into a mobile app.

For other life-log data, participants measured their body weight using their own devices. Physical activity, number of steps taken per day, and sleep time were measured using Fitbit devices. Salt intake was estimated using a GENEN monitor (Kono ME Laboratory, Inc.), a validated tool for assessing daily salt consumption [19]. Participants were encouraged to log these data consistently, enabling healthcare professionals to monitor trends and provide tailored recommendations through the apps.

The demographic and clinical characteristics were recorded at the start of the program. These variables included age, sex, and body mass index (BMI). Additionally, medical history and treatment information were recorded, including diagnoses of hypertension, dyslipidemia, diabetes mellitus, coronary artery disease, and stroke, as well as ongoing medication use. The most recent health checkup results were also obtained, including blood pressure data before the Mystar program, serum lipid levels, and HbA1c levels from blood tests.

Data on app usage were collected, including session count, time spent per session, time spent inputting lifelog data, time spent viewing lifelog data, time spent viewing the home screen, time spent viewing the study materials, time spent using the chat function, chat volume, and meal photo count. The session count was defined as the average number of app launches per day. The time spent viewing lifelog data indicates the duration within the total usage time during which the participants viewed the visualized graphs or summaries of their logged data. The time spent viewing the home screen indicated the time participants spent viewing lifestyle goals and interview information. The app counted the chat volume and meal photo count as average numbers per week. For modeling, these log-derived metrics were aggregated over the observation window to represent the frequency and intensity of engagement with the program. This study categorized body weight, physical activity (step count), sleep duration, estimated salt intake, and app usage metrics as behavioral variables for modeling.

### Statistical analysis

**Fig. 1:**
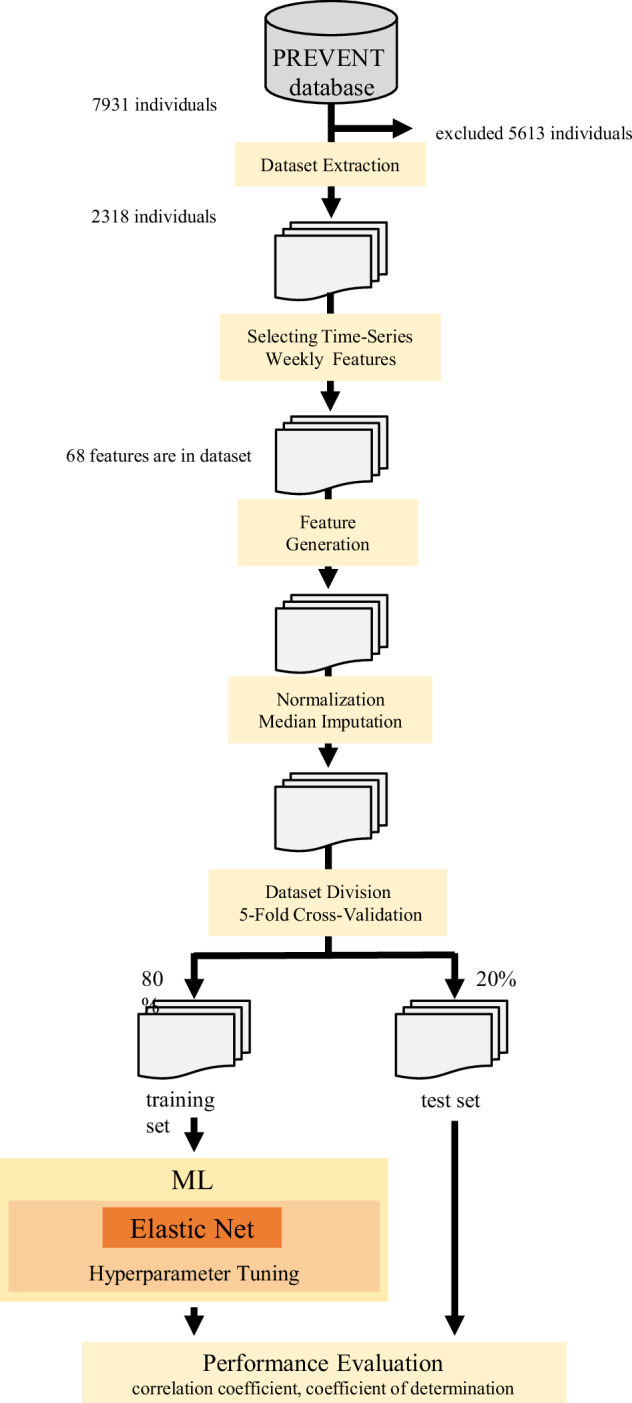
Workflow for feature engineering and model development. Schematic overview of the analytic workflow used to evaluate the predictive utility of feature-engineered variables for changes in systolic blood pressure. Data were extracted from the PREVENT database and structured into weekly time-series features. Featuretools was used to generate additional predictors through automated feature engineering. After normalization and median imputation, the dataset was split into training and test sets using a five-fold cross-validation. ElasticNet regression was applied with hyperparameter tuning using Optuna. Prediction models were developed at 4, 8, 12, and 22 weeks using only data available up to each time point. The model performance was evaluated by comparing the correlation coefficients and coefficients of determination between the models with and without feature-engineered variables

The overall workflow of the analysis is illustrated in Fig. 1. Continuous variables are presented as mean ± SD, and categorical variables are presented as counts and percentages. For participants who completed the program before 24 weeks, the mean blood pressure from the final recorded week was used as the endpoint.

### Development of SBP change prediction model

To ensure the robustness of the prediction models and minimize the influence of potential outliers, participants whose SBP changes fell within the top or bottom 2.5% of the distribution were excluded from the analysis. These extreme changes may reflect unmeasured factors, such as substantial modifications in antihypertensive therapy during the program, which could confound the relationship between program-related variables and SBP outcomes.

To evaluate the predictive value of feature-engineered predictors for SBP changes, we compared two modeling approaches [1]: a model using only conventional predictors (e.g., demographic, clinical, and behavioral variables] and [2] a model incorporating additional variables generated through automated feature engineering. Features were generated using FeatureTools [20], a Python-based library that automatically extracts informative variables from time-series datasets. At each prediction point (4, 8, 12, and 22 weeks), models were built to estimate the change in SBP from the baseline. Only data available up to each specific time point (e.g., 4-week data for 4-week prediction) were used to construct predictors, and no future data beyond the prediction time point were included. The predictors included baseline clinical data, changes from baseline to the target week, and weekly average lifestyle and app usage metrics for the corresponding week.

The dataset was divided using a five-fold cross-validation. For each outer fold, the data were first split into a training-validation set and an independent test set. The training-validation set was then further divided into a training set and a validation set using a 4:1 ratio to perform hyperparameter tuning. Normalization and median imputation were applied to all continuous variables before model training. ElasticNet regression was used to train all the models [21]. This regularized regression method combines L1 and L2 penalties, balancing predictive performance and interpretability. The hyperparameter optimization for ElasticNet was performed using Optuna [22], which employs a tree-structured Parzen estimator (TPE) to efficiently search the parameter space. No stepwise feature elimination was performed; instead, the model performance was directly optimized through hyperparameter tuning.

After tuning, the final models were retrained using only the training subset and evaluated on the held-out test set. The model performance was assessed based on the Pearson correlation coefficient and the coefficient of determination (R²) between the predicted and observed SBP changes. The final evaluation metrics were reported as averages across all cross-validation folds. All analyses were performed using Python software (ver. 3.13.1) using the following packages: Pandas (2.2.3), scikit-learn (1.6.0), feature tools (1.31.0), and Optuna (4.1.0).

## Results

During the study period, 6699 individuals completed the 24-week Mystar program. Of these, 1227 lacked SBP data at either the start or end of the program, and 3030 had insufficient weekly morning SBP measurements. An additional 124 participants with extreme SBP changes were excluded from the study. Consequently, 2318 participants were included in the analysis Table 1. The mean age of the participants was 56.7 years, and most were male (86.6%). Most patients had hypertension (81.1%), and 64.2% were prescribed at least one antihypertensive agent. The average systolic and diastolic blood pressure at the start of the program were 129.7 mmHg and 84.0 mmHg, respectively. Information related to app usage is presented in Supplementary Table 1.

**Table 1:** Participants characteristics

	*n* = 2318
Age (years)	56.7 (6.7)
Gender, *n* (%)	
Male	2007 (86.6)
Female	311 (13.4)
Disease conditions	
Hypertension, *n* (%)	1880 (81.1)
Diabetes mellitus, *n* (%)	932 (40.2)
Dyslipidemia, *n* (%)	1355 (58.5)
Previous stroke, *n* (%)	113 (4.9)
Previous ischemic heart disease, *n* (%)	179 (7.7)
Medication	
Antihypertensive agent	
0, *n* (%)	831 (35.8)
1, *n* (%)	653 (28.2)
≥2, *n* (%)	834 (36.0)
Cholesterol-lowering agent	
0, *n* (%)	1385 (59.7)
1, *n* (%)	831 (35.8)
≥2, *n* (%)	102 (4.5)
Oral antidiabetic agent	
0, *n* (%)	1693 (73.0)
1, *n* (%)	199 (8.6)
≥2, *n* (%)	426 (18.4)
Insulin injection	46 (2.0)
Condition at start of program	
BMI (kg/m^2^), *n* = 2302	26.8 (4.3)
Systolic blood pressure (mmHg)	129.7 (11.1)
Diastolic blood pressure (mmHg)	84.0 (8.8)
HbA1c (%), *n* = 2178	6.3 (1.0)
L/H ratio (%), *n* = 2224	2.3 (0.8)
eGFR (mL/min/1.73m^2^), *n* = 2161	74.1 (14.1)

*L/H ratio* low-density lipoprotein cholesterol/high-density lipoprotein cholesterol, *eGFR* estimated Glomerular Filtration Rate. Values are shown as mean (standard deviation) or as ordinal variables and counts (%) for categorical variables

**Fig. 2:**
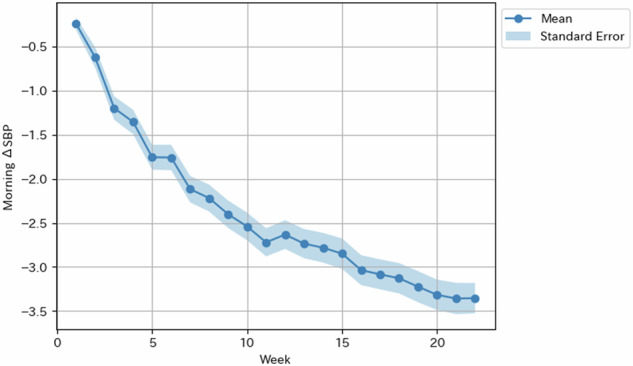
Weekly changes in the mean systolic blood pressure from baseline

Figure 2 shows the weekly changes in mean SBP from the start of the program. SBP gradually decreased throughout the 24-week Mystar program, with a mean reduction of 3.5 mmHg by the end of the program.

**Fig. 3:**
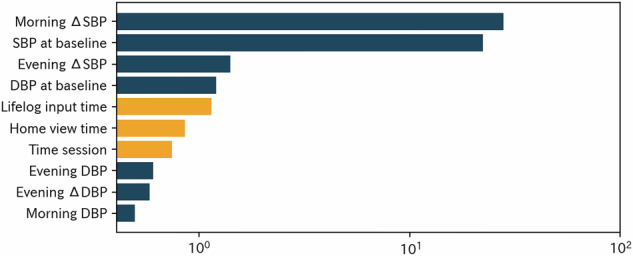
Feature importance rankings at 4 weeks. Bar plot showing the top 10 predictors of systolic blood pressure (SBP) change at 4 weeks based on feature importance. Dark blue bars represent blood pressure-related variables such as morning and evening SBP changes and baseline blood pressure values. Orange bars represent app usage metrics, including time spent inputting lifelog data, viewing the home screen, and per session Feature importance is plotted on a log scale because the distribution of importance values is highly right-skewed, and log transformation improves interpretability without altering relative rankings

**Fig. 4:**
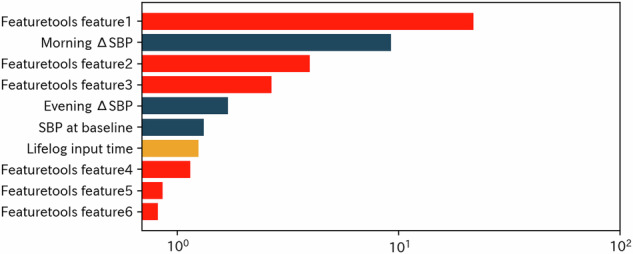
Feature importance rankings after feature engineering at 4 weeks. Bar plot showing the top 10 predictors of systolic blood pressure (SBP) change at 4 weeks after applying feature engineering with feature tools. The red and dark blue bars represent newly generated composite features and original blood pressure-related variables, respectively. The orange bars represent app usage metrics. Feature importance is plotted on a log scale because the distribution of importance values is highly right-skewed, and log transformation improves interpretability without altering relative rankings. Top engineered features include: • Featuretools feature1 = (SBP at baseline)^2^ / (Morning SBP). • Featuretools feature2 = (SBP at baseline)^2^ × (DBP at baseline) / (Morning SBP). • Featuretools feature3 = (Morning SBP) / (SBP at baseline)^2^. • Featuretools feature4 = (SBP at baseline)^2^ / (Evening SBP). • Featuretools feature5 = (Morning DBP) / [(SBP at baseline)^2^ × (DBP at baseline)]. • Featuretools feature6 = (Evening SBP) / (SBP at baseline)^2^

Figure 3 summarizes the feature importance rankings at four weeks, representing early phase prediction. The top-ranked features included morning and baseline SBP changes. App usage metrics, such as the time spent viewing lifelog data and time spent on the home screen, also emerged among the top 10 features after feature engineering. Similar trends were observed at 8, 12, and 22 weeks (Supplementary Figs. 1–3), although the relative importance of app engagement indicators tended to decline over time as recent BP values became stronger predictors. Following feature engineering with feature tools, several new composite features replaced many of the top original predictors by week four (Fig. 4). Although the original BP features remained influential, the composite predictors were highly ranked. This pattern persisted at weeks 8 and 12; however, by week 22, BP-related features regained predominance in the importance rankings (Supplementary Figs. 4–6).

We further examined the correlations between the predictors and changes in SBP. At week 4, the top engineered feature showed a stronger correlation (r = 0.561) than the best original predictor (r = 0.455) (Supplementary Table 2). Similar differences were observed in week 8. However, by week 22, the correlation coefficients between the engineered and original features were comparable (Supplementary Table 2).

The predictive performance of ElasticNet is shown in Table 2. Both models, those with and without feature-engineered predictors, demonstrated similar performance across all time points. At week 4, the correlation coefficients were 0.561 (without feature engineering) and 0.559 (with feature engineering), and comparable results were observed at weeks 8 and 12. By week 22, both models achieved a high correlation of approximately 0.85. The corresponding R² values remained similar across all the time points, with only minor differences between the models throughout the 22-week period.

**Table 2:** Prediction performance using ElasticNet

Time Point	Without Feature Engineering		With Feature Engineering	
	r [95%CI]	R^2^ [95%CI]	r [95%CI]	R^2^ [95%CI]
4w	0.561 [0.540, 0.581]	0.311 [0.290, 0.332]	0.559 [0.538, 0.581]	0.311 [0.288, 0.334]
8w	0.622 [0.595, 0.650]	0.385 [0.351, 0.418]	0.619 [0.587, 0.650]	0.379 [0.340, 0.418]
12w	0.665 [0.637, 0.694]	0.438 [0.400, 0.476]	0.674 [0.662, 0.686]	0.451 [0.435, 0.467]
22w	0.853 [0.839, 0.867]	0.726 [0.702, 0.749]	0.854 [0.842, 0.867]	0.728 [0.707, 0.749]

95%CI 95% confidence interval

## Discussion

This study evaluated feature-engineered predictors of SBP changes over 24 weeks using data from the mHealth-based disease management program Mystar. The three main strengths of the study were: (1) the integration of behavioral variables into predictive models; (2) late-phase models achieved high accuracy, largely driven by recent BP data; and (3) the use of feature engineering to generate composite predictors with modestly stronger associations with SBP change during the early phase (weeks 4 and 8). Despite these strengths, the overall performance of the ElasticNet models remained largely comparable, regardless of whether feature engineering was applied.

To contextualize these findings, we examined how our modeling results aligned with the prior literature on BP predictions. This study incorporated behavioral variables into predictive modeling to improve SBP change prediction through feature engineering. However, prior research has consistently shown that behavioral metrics have limited associations with BP regulation [9, 14]. Our previous study found that app usage time had a limited impact on predicting blood pressure changes [9]. In another study, Majahalme et al. demonstrated that short-term BP records were more predictive than cumulative behavioral patterns [23]. Together, these findings highlight the clinical importance of continuously monitoring BP dynamics, not only for improving short-term prediction accuracy but also for detecting early phases of inadequate hypertension control. This has also been emphasized in studies linking BP variability with long-term cardiovascular risk [24]. Accordingly, we focused on quantifying the early-phase incremental information from interpretable, feature-engineered predictors and documenting how their contributions shift over time in real-world data aggregated at the weekly level.

Our findings also revealed that digital behavioral indicators, such as the time spent viewing lifelog data, were among the top 10 predictors in the models. These digital indicators, passively derived from user activity logs, may reflect underlying engagement patterns with physiological relevance [14, 25]. Their predictive utility is particularly evident during the initial phase of lifestyle intervention. The integration of such engagement metrics was suggested to be effective in stratifying users who were less responsive to blood pressure reduction during the early phase of the program. Furthermore, mHealth platforms capable of detecting and interpreting these behavioral patterns may facilitate the early identification of individuals requiring personalized support. In practical terms, for participants showing an early decline in engagement, such signals could be used to trigger app-use reminders and reinforce data entry, and to assign more intensive phone-based counseling within the routine schedule, or to schedule periodic chat-based follow-up to confirm adherence and summarize individualized advice. These capabilities have the potential to complement traditional BP monitoring approaches and contribute to more comprehensive hypertension management strategies [26, 27].

Although machine learning models often require large datasets for optimal performance [28], our findings suggest that feature engineering can generate informative predictors even when early phase data are limited. In this study, composite features derived through simple mathematical operations provided modest gains in the early phases. This illustrates the utility of automated interpretable feature engineering when data are limited. Overall, the prediction performance of ElasticNet remained largely comparable regardless of whether feature engineering was applied. These findings underscore the importance of balancing transparency and complexity when developing predictive models for healthcare applications. In this study, we deliberately employed ElasticNet with simple composite features to emphasize interpretability and model transparency, thereby providing clinically intuitive insight into how individual predictors contribute to SBP change. Although this choice inevitably constrains the capacity to represent complex nonlinear and higher-order interactions, the modest but consistent early-phase signals identified in this work support the value of starting from an interpretable modeling framework in mHealth-based hypertension management. Future work may incorporate more flexible approaches in a complementary role, while preserving interpretability and operational simplicity, to further elucidate the multifactorial, time-varying processes underlying BP regulation.

We initially hypothesized that feature engineering would uncover novel lifestyle-derived predictors, such as body weight, step count, and salt intake. However, the top-performing predictors were primarily composite features derived from early BP measurements. This may be due to the limited temporal granularity of the behavioral variables, which were mostly aggregated as weekly averages or cumulative changes in the data, a format that likely smooths short-term lifestyle fluctuations that could be coupled with day-to-day BP changes and therefore may lead to a conservative estimate of the behavioral contribution. Future iterations of the mHealth program that collect higher-frequency behavioral inputs could better clarify how engagement and lifestyle factors interact with BP dynamics. In addition, restricting feature generation to basic mathematical transformations likely limits the detection of complex interactions. Moreover, given that BP regulation is influenced by multifactorial and time-varying factors [29], predicting long-term BP changes from early phase data (e.g., at week 4) might have been particularly challenging in the context of this study.

In contrast, the predictive performance improved by week 22, when cumulative behavioral and physiological data were available, enabling more accurate forecasts of near-future BP trends (e.g., at 24 weeks). This suggests that more recent data offer greater predictive utility for modeling future outcomes. Accordingly, we regard this late-phase performance as a benchmark rather than a source of novelty in this study. Although direct comparisons are limited by differences in prediction targets, horizons, and evaluation metrics, several digital-health BP prediction studies report broadly comparable performance levels. For example, machine-learning models predicting treatment response in a digital-therapeutic program achieved area under the receiver operating characteristic curve (AUROC) values of 0.82 for ≥10-mmHg systolic BP reduction and 0.69 for categorical BP improvement [14]. Similarly, another study applying random forest models to predict intervention completion in a mobile hypertension program reported an AUROC of 0.78 [30]. In addition, long short-term memory- and random-forest–based models using mobile phone and wearable-sensor data have demonstrated high accuracy for BP forecasting, including 95.0% accuracy for SBP and 92.5% for DBP [11]. Together, these studies indicate that our late-phase accuracy (r ≈ 0.85) aligns with the upper range of performance observed across digital BP estimation and forecasting frameworks, even though methodological heterogeneity precludes direct one-to-one comparison.

In this light, we emphasize the present work’s novelty in quantifying the modest early-phase incremental signal from interpretable, feature-engineered and engagement-related variables under real-world weekly aggregation, rather than in the late-phase benchmark itself. Given that we did not assess the cost-effectiveness or health-system implementation burden of adaptive prediction strategies, future work will need to evaluate resource implications, scalability, and economic viability [31]. Building on this observation, a practical strategy may involve implementing a short-term layered prediction approach in which BP changes are sequentially predicted in 2-week increments (e.g., weeks 4 to 6 and 6 to 8). Such a framework would allow adaptive updating of the prediction model using the most recent engagement and BP data, progressively improving accuracy over time [32]. Furthermore, providing users with short-term predictions and feedback may enhance their motivation and engagement, thereby supporting sustainable behavioral changes within mHealth-based hypertension management programs.

### Limitation

This study had several limitations. First, we used data from a specific mHealth-based disease management program, and several characteristics of the dataset introduce inherent limitations. The analytic sample consisted only of participants who completed the 24-week program with sufficient longitudinal data, and the proportion of male participants was markedly high. In addition, changes in antihypertensive medication during the program were recorded only through participant self-report, so treatment modifications may not have been captured with complete accuracy. These factors may introduce selection bias and constrain the applicability of the results beyond this specific user group. Validation using external datasets across diverse settings is necessary to assess the robustness of the results. Second, when feature engineering was applied, the transformations were intentionally limited to simple mathematical operations to preserve interpretability. This restriction may have limited the ability of the model to capture nonlinear or higher-order interactions between the variables. Third, the use of ElasticNet regression may have further constrained the ability to model nonlinear interactions. Fourth, because blood pressure shows substantial diurnal and weekly variability, prior studies recommend averaging home BP over about one week for reliable assessment [33, 34]. In this study, however, behavioral and engagement indicators were aggregated weekly, which may have smoothed short-term fluctuations and weakened their within-week association with morning SBP. Future research should determine whether using higher temporal-resolution data can improve prediction while preserving interpretability and minimizing participant burden. Lastly, participants were drawn from the general population enrolled in the Mystar program, rather than from a cohort with poorly controlled hypertension. Consequently, the magnitude of BP change was relatively modest, potentially attenuating the observed predictive associations. Future studies may benefit from incorporating higher-frequency data and more targeted populations to refine predictive performance and enhance clinical relevance.

### Perspective of Asia

Hypertension management in Asia faces a high burden and region-specific risks (e.g., high salt intake and morning hypertension), and scalable mHealth programs (including workplace and insurer-supported models) may benefit from interpretable prediction using routinely collected home BP and engagement logs to flag early risk of suboptimal SBP response when visit-based follow-up is limited. In our weekly-aggregated real-world data, recent home BP dominated long-term SBP-change prediction while early engagement signals added only modest information; future work should test generalizability across Asian settings and integrate these predictors into guideline-aligned remote care without increasing participant burden [27, 29, 31, 33, 34].

## Conclusion

This study investigated the predictive utility of feature-engineered variables for SBP changes using an mHealth-based disease management program. Although feature engineering improved the early phase correlations between individual predictors and SBP change, it did not lead to a meaningful improvement in the overall prediction performance of the ElasticNet model. Behavioral indicators such as app engagement metrics were identified as important predictors in the initial phase; however, their relative contributions diminished over time as recent BP values became more influential. Early-phase monitoring that integrates short-term BP patterns with engagement indicators enabled reasonably accurate estimation of end-of-program SBP, and these findings suggest that such early predictions may be useful for identifying participants at risk of suboptimal response and for triggering timely, individualized guidance and proactive adjustment of intervention intensity within mHealth programs. Further studies are required to validate these results and assess their applicability in broader clinical contexts.

## Supplementary information

Supplementary figure

Supplementary table

## Data Availability

The datasets generated and analyzed in this study are available from the corresponding author upon reasonable request.

## References

[1] Kjeldsen SE. Hypertension and cardiovascular risk: general aspects. Pharm Res. 2018;129:95–9. 10.1016/j.phrs.2017.11.003 29127059

[2] Fuchs FD, Whelton PK. High blood pressure and cardiovascular disease. Hypertension. 2020;75:285–92. 31865786 10.1161/HYPERTENSIONAHA.119.14240 PMC10243231

[3] Webb AJS, Werring DJ. New insights into cerebrovascular pathophysiology and hypertension. Stroke. 2022;53:1054–64. 35255709 10.1161/STROKEAHA.121.035850 PMC7615037

[4] Mills KT, Stefanescu A, He J. The global epidemiology of hypertension. Nat Rev Nephrol. 2020;16:223–37. 32024986 10.1038/s41581-019-0244-2 PMC7998524

[5] Dai H, Bragazzi NL, Younis A, Zhong W, Liu X, Wu J, et al. Worldwide trends in prevalence, mortality, and disability-adjusted life years for hypertensive heart disease from 1990 to 2017. Hypertension. 2021;77:1223–33. 33583201 10.1161/HYPERTENSIONAHA.120.16483

[6] Carey RM, Wright JT Jr, Taler SJ, Whelton PK. Guideline-driven management of hypertension: an evidence-based update. Circ Res. 2021;128:827–46. 33793326 10.1161/CIRCRESAHA.121.318083 PMC8034801

[7] Shimbo D, Artinian NT, Basile JN, Krakoff LR, Margolis KL, Rakotz MK, et al. Self-measured blood pressure monitoring at home: A joint policy statement from the American Heart Association and American Medical Association. Circulation. 2020;142:e42–63. 32567342 10.1161/CIR.0000000000000803

[8] Schorr EN, Gepner AD, Dolansky MA, Forman DE, Park LG, Petersen KS, et al. Harnessing mobile health technology for secondary cardiovascular disease prevention in older adults: A scientific statement from the American Heart Association. Circ Cardiovasc Qual Outcomes. 2021;14:e000103. 33793309 10.1161/HCQ.0000000000000103

[9] Kanai M, Toda T, Yamamoto K, Akimoto M, Hagiwara Y. A mobile health-based disease management program improves blood pressure in people with multiple lifestyle-related diseases at risk of developing vascular disease ― A retrospective observational study. Circ Rep. 2022;4:322–29. 35860354 10.1253/circrep.CR-22-0024 PMC9257458

[10] Koshimizu H, Kojima R, Kario K, Okuno Y. Prediction of blood pressure variability using deep neural networks. Int J Med Inform 2020; 136. 10.1016/j.ijmedinf.2019.104067 31955052

[11] Xiang Y, Li S, Zhang P. An exploration in remote blood pressure management: application of daily routine pattern based on mobile data in health management. Fundam Res. 2022;2:154–65. 38933904 10.1016/j.fmre.2021.11.006 PMC11197610

[12] Janjua ZH, Kerins D, O’Flynn B, Tedesco S. Knowledge-driven feature engineering to detect multiple symptoms using ambulatory blood pressure monitoring data. Comput Methods Prog Biomed. 2022;217:106638. 10.1016/j.cmpb.2022.106638 35220199

[13] Tsoi KKF, Chan NB, Yiu KKL, Poon SKS, Lin B, Ho K. Machine learning clustering for blood pressure variability applied to systolic blood pressure intervention trial (Sprint) and the Hong Kong community cohort. Hypertension. 2020;76:569–76. 32594794 10.1161/HYPERTENSIONAHA.119.14213

[14] Guthrie NL, Carpenter J, Edwards KL, Appelbaum KJ, Dey S, Eisenberg DM, et al. Emergence of digital biomarkers to predict and modify treatment efficacy: machine learning study. BMJ Open. 2019;9:e030710. 31337662 10.1136/bmjopen-2019-030710 PMC6661657

[15] Parati G, Ochoa JE, Lombardi C, Bilo G. Assessment and management of blood-pressure variability. Nat Rev Cardiol. 2013;10:143–55. 23399972 10.1038/nrcardio.2013.1

[16] Izzo R, Mancusi C, De Stefano G, Albano G, Losi MA, Trimarco V, et al. Achievement of target SBP without attention to decrease in DBP can increase cardiovascular morbidity in treated arterial hypertension: the Campania Salute Network. J Hypertens. 2019;37:1889–97. 31205199 10.1097/HJH.0000000000002128

[17] Miki T, Yamada J, Ishida S, Sakui D, Kanai M, Hagiwara Y. Exploring the feasibility and initial impact of an mHealth-based disease management program for chronic ischemic heart disease: formative study. JMIR Form Res. 2024;8:e56380. 39173150 10.2196/56380 PMC11377902

[18] Campbell NRC, Paccot Burnens M, Whelton PK, Angell SY, Jaffe MG, Cohn J, et al. 2021 World Health Organization guideline on pharmacological treatment of hypertension: policy implications for the region of the Americas. Lancet Reg Health Am. 2022; 9. 10.1016/j.lana.2022.100219 PMC9107389 35711684

[19] Yamasue K, Tochikubo O, Kono E, Maeda H. Self-monitoring of home blood pressure with estimation of daily salt intake using a new electrical device. J Hum Hypertens. 2006;20:593–98. 16710288 10.1038/sj.jhh.1002049

[20] PyPI [Internet]. Accessed 2025 Apr 10. featuretools. https://pypi.org/project/featuretools/.

[21] De Mol C, De Vito E, Rosasco L. Elastic-net regularization in learning theory. J Complex. 2009;25:201–30.

[22] Akiba T, Sano S, Yanase T, Ohta T, Koyama M. Optuna. In Proceedings of the 25th ACM SIGKDD International Conference on Knowledge Discovery & Data Mining [Internet], 2019. ACM: New York, NY.

[23] Majahalme S, Turjanmaa V, Weder AB, Lu H, Tuomisto MT, Uusitalo A. Blood pressure level and variability in the prediction of blood pressure after 5-year follow-up. Hypertension. 1996;28:725–31. 8901815 10.1161/01.hyp.28.5.725

[24] Chowdhury EK, Wing LMH, Jennings GLR, Beilin LJ, Reid CM. ANBP2 Management Committee. Visit-to-visit (long-term) and ambulatory (short-term) blood pressure variability to predict mortality in an elderly hypertensive population. J Hypertens. 2018;36:1059–67. 29266060 10.1097/HJH.0000000000001652

[25] Hartin PJ, Nugent CD, McClean SI, Cleland I, Tschanz JT, Clark CJ, et al. The empowering role of mobile apps in behavior change interventions: the gray matters randomized controlled trial. JMIR MHealth UHealth. 2016;4:e93. 27485822 10.2196/mhealth.4878 PMC4987494

[26] Bryant KB, Sheppard JP, Ruiz-Negrón N, Kronish IM, Fontil V, King JB, et al. Impact of self-monitoring of blood pressure on processes of hypertension care and long-term blood pressure control. J Am Heart Assoc. 2020;9:e016174. 32696695 10.1161/JAHA.120.016174 PMC7792261

[27] Kario K. Management of hypertension in the digital era: small wearable monitoring devices for remote blood pressure monitoring. Hypertension. 2020;76:640–650. 32755418 10.1161/HYPERTENSIONAHA.120.14742 PMC7418935

[28] Rajput D, Wang WJ, Chen CC. Evaluation of a decided sample size in machine learning applications. BMC Bioinforma. 2023;24:48. 10.1186/s12859-023-05156-9 PMC9926644 36788550

[29] Parati G, Bilo G, Kollias A, Pengo M, Ochoa JE, Castiglioni P, et al. Blood pressure variability: methodological aspects, clinical relevance and practical indications for management - a European Society of Hypertension position paper ∗. J Hypertens. 2023;41:527–44. 36723481 10.1097/HJH.0000000000003363

[30] Guthrie NL, Berman MA, Edwards KL, Appelbaum KJ, Dey S, Carpenter J, et al. Achieving rapid blood pressure control with digital therapeutics: Retrospective cohort and machine learning study. JMIR Cardio. 2019;3:e13030. 31758792 10.2196/13030 PMC6834235

[31] Nomura A, Tanigawa T, Kario K, Igarashi A. Cost-effectiveness of digital therapeutics for essential hypertension. Hypertens Res. 2022;45:1538–48. 35726085 10.1038/s41440-022-00952-x PMC9474296

[32] Wang G, Zhou S, Rezaei S, Liu X, Huang A. An ambulatory blood pressure monitor mobile health system for early warning for stroke risk: longitudinal observational study. JMIR MHealth UHealth. 2019;7:e14926. 31670694 10.2196/14926 PMC6913731

[33] Lin HJ, Pan HY, Chen CH, Cheng HM, Chia YC, Sogunuru GP, et al. Standardized home blood pressure monitoring: Rationale behind the 722 protocol. J Clin Hypertens. 2022;24:1161–73. 10.1111/jch.14549 PMC9532917 36196472

[34] Morris EC, Tucker KL, McManus RJ, Stevens RJ. The importance of experience: insights into optimal home-blood pressure monitoring regimens from the TASMINH4 Trial. J Hypertens. 2025;43:1400–6. 40407130 10.1097/HJH.0000000000004062 PMC12237135

