# Supplementary material for: Investigating feature-engineered predictors for systolic blood pressure changes in an mHealth-based disease management program
Source: Hypertens Res. 2026 Feb 17;49(4):1204–13. doi: 10.1038/s41440-026-02569-w (PMC13050640; doi:10.1038/s41440-026-02569-w)
Supplement: Supplementary file 1 — Supplementary figure [file 41440_2026_2569_MOESM1_ESM.pdf]

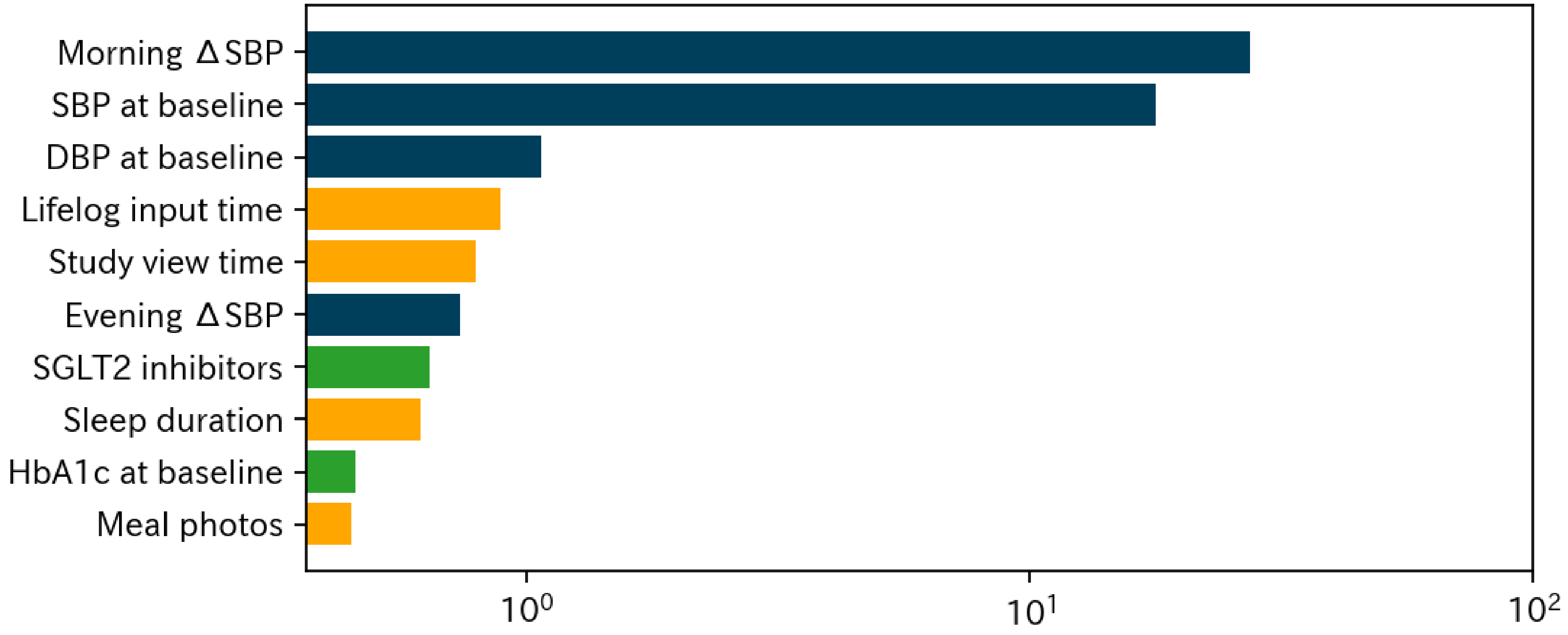

Supplementary Figure 1. Feature importance rankings at 8 weeks.
Bar plot showing the top 10 predictors of systolic blood pressure change at 8 weeks based on feature importance. Dark blue bars represent blood pressure-related variables. Orange bars represent app usage metrics. Green bars represent baseline participant's information. Feature importance is plotted on a log scale because the distribution of importance values is highly right-skewed, and log transformation improves interpretability without altering relative rankings.

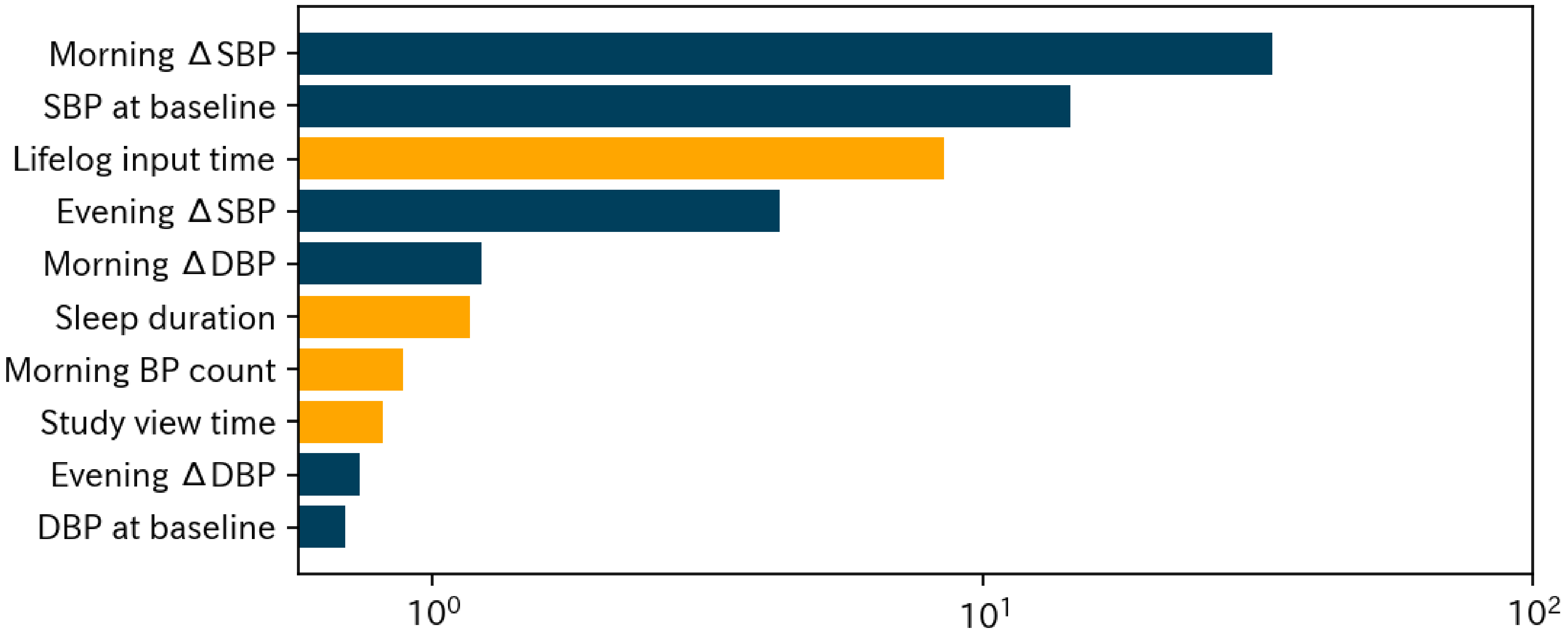

Supplementary Figure 2. Feature importance rankings at 12 weeks.
Bar plot showing the top 10 predictors of systolic blood pressure change at 8 weeks based on feature importance. Dark blue bars represent blood pressure-related variables. Orange bars represent app usage metrics. Feature importance is plotted on a log scale because the distribution of importance values is highly right-skewed, and log transformation improves interpretability without altering relative rankings.

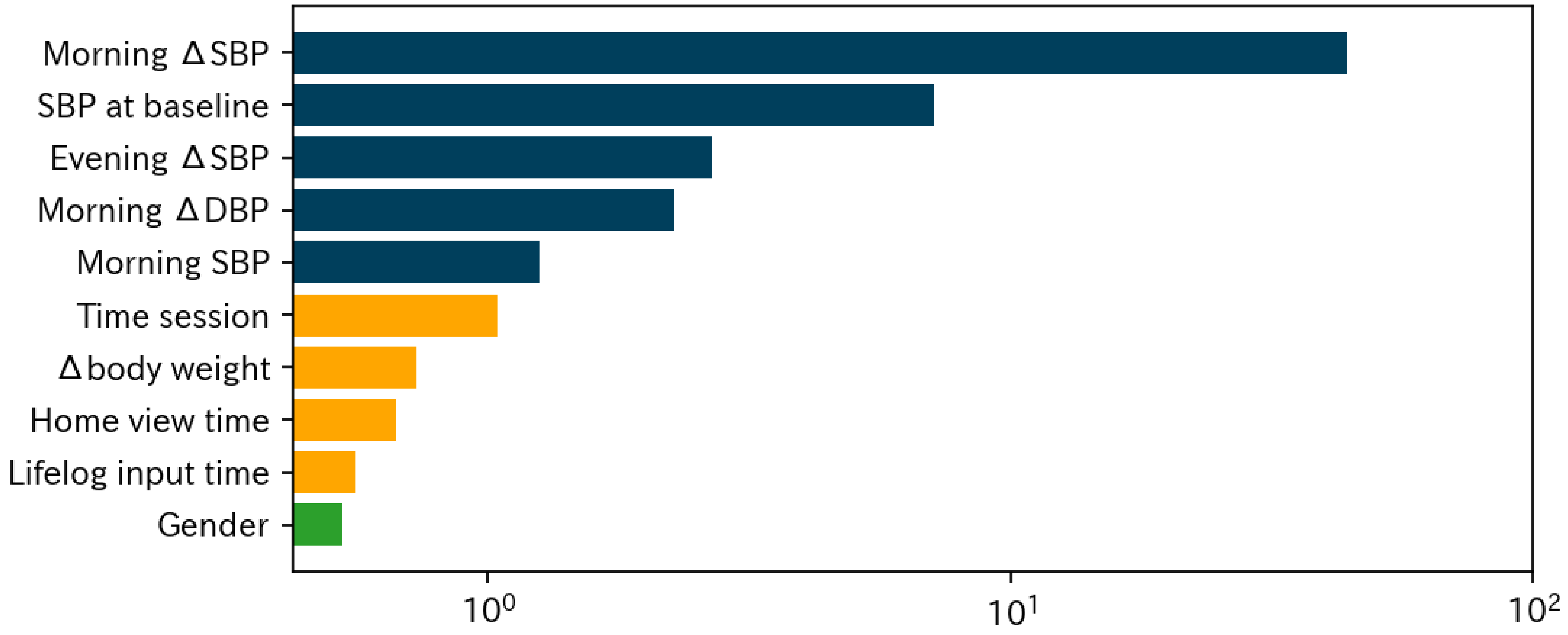

Supplementary Figure 3. Feature importance rankings at 22 weeks.

Bar plot showing the top 10 predictors of systolic blood pressure change at 22 weeks based on feature importance. Dark blue bars represent blood pressure-related variables. Orange bars represent app usage metrics. Green bar represents baseline participant's information. Feature importance is plotted on a log scale because the distribution of importance values is highly right-skewed, and log transformation improves interpretability without altering relative rankings.

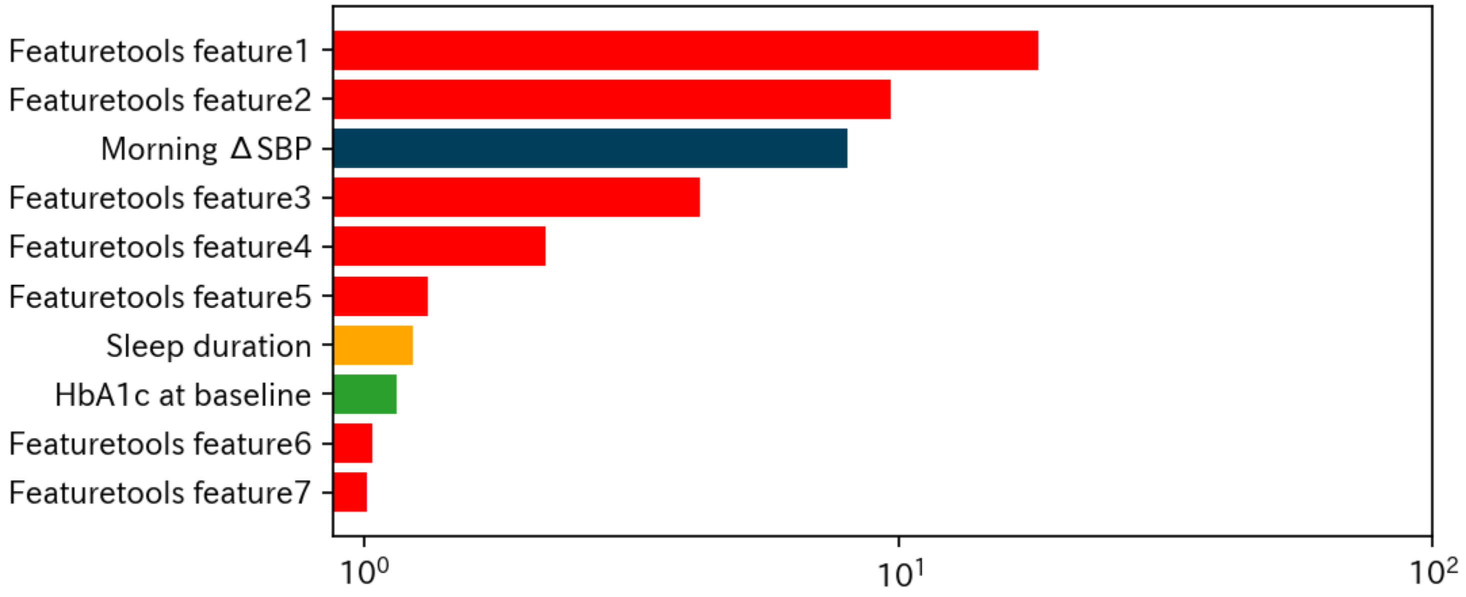

Supplementary Figure 4. Feature importance rankings after feature engineering at 8 weeks.
Bar plot showing the top 10 predictors of systolic blood pressure (SBP) change at 8 weeks after applying feature engineering with feature tools. The red and dark blue bars represent newly generated composite features and original blood pressure-related variables, respectively. Orange bars represent app usage metrics. Green bar represents baseline participant's information. Feature importance is plotted on a log scale because the distribution of importance values is highly right-skewed, and log transformation improves interpretability without altering relative rankings.
Top engineered features include:

- Featuretools feature1 = $(\text{SBP at baseline})^2$ / (Morning SBP)
- Featuretools feature2 = (DBP at baseline) × (Morning ΔSBP)
- Featuretools feature3 = (Morning ΔSBP) × $(\text{SBP at baseline})^2$
- Featuretools feature4 = $(\text{SBP at baseline})^2$ × (DBP at baseline) / (Morning SBP)
- Featuretools feature5 = $(\text{SBP at baseline})^2$ / $(\text{Morning SBP})^2$
- Featuretools feature6 = (Morning SBP) × (Evening SBP) / $(\text{SBP at baseline})^2$
- Featuretools feature7 = (Morning SBP) / $(\text{SBP at baseline})^2$

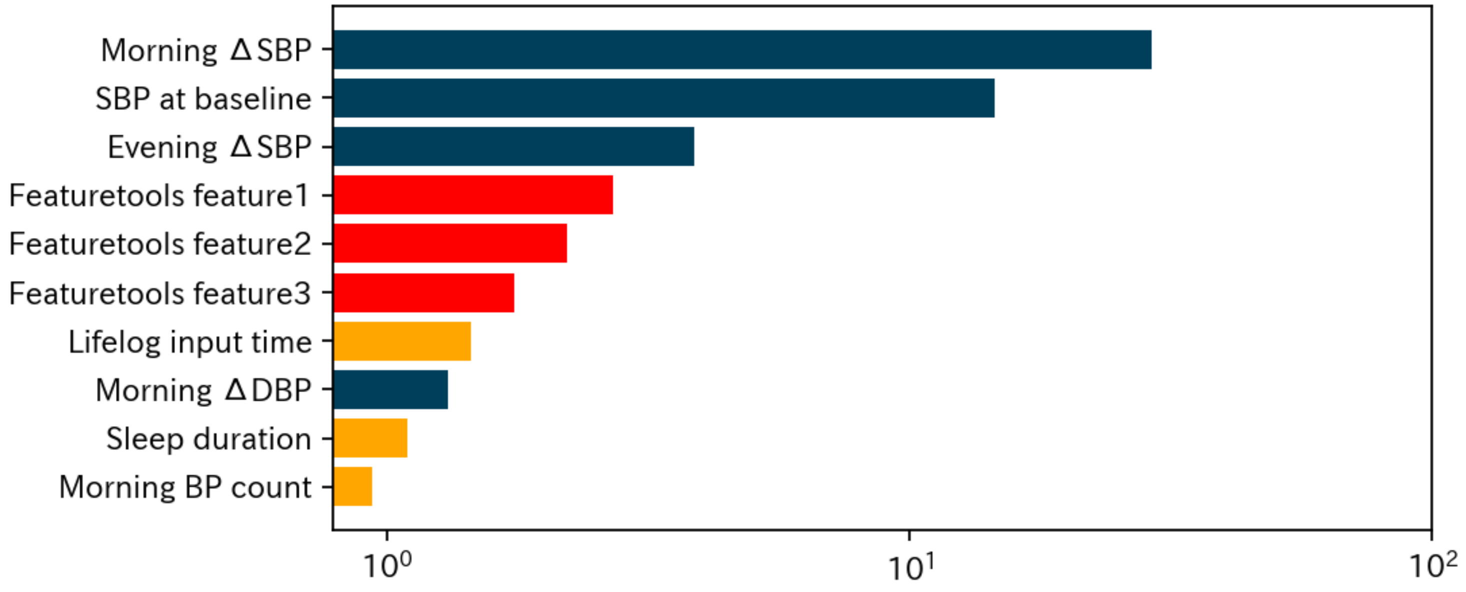

Supplementary Figure 5. Feature importance rankings after feature engineering at 12 weeks.
Bar plot showing the top 10 predictors of systolic blood pressure (SBP) change at 12 weeks after applying feature engineering with feature tools. The red and dark blue bars represent newly generated composite features and original blood pressure-related variables, respectively. Orange bars represent app usage metrics. Feature importance is plotted on a log scale because the distribution of importance values is highly right-skewed, and log transformation improves interpretability without altering relative rankings.
Top engineered features include:

- Featuretools feature1 = (Morning ΔSBP) × (Evening BP count)
- Featuretools feature2 = (Morning ΔSBP) × (SBP at baseline)^2 × (Session count)
- Featuretools feature3 = (Morning ΔSBP) × (SBP at baseline)

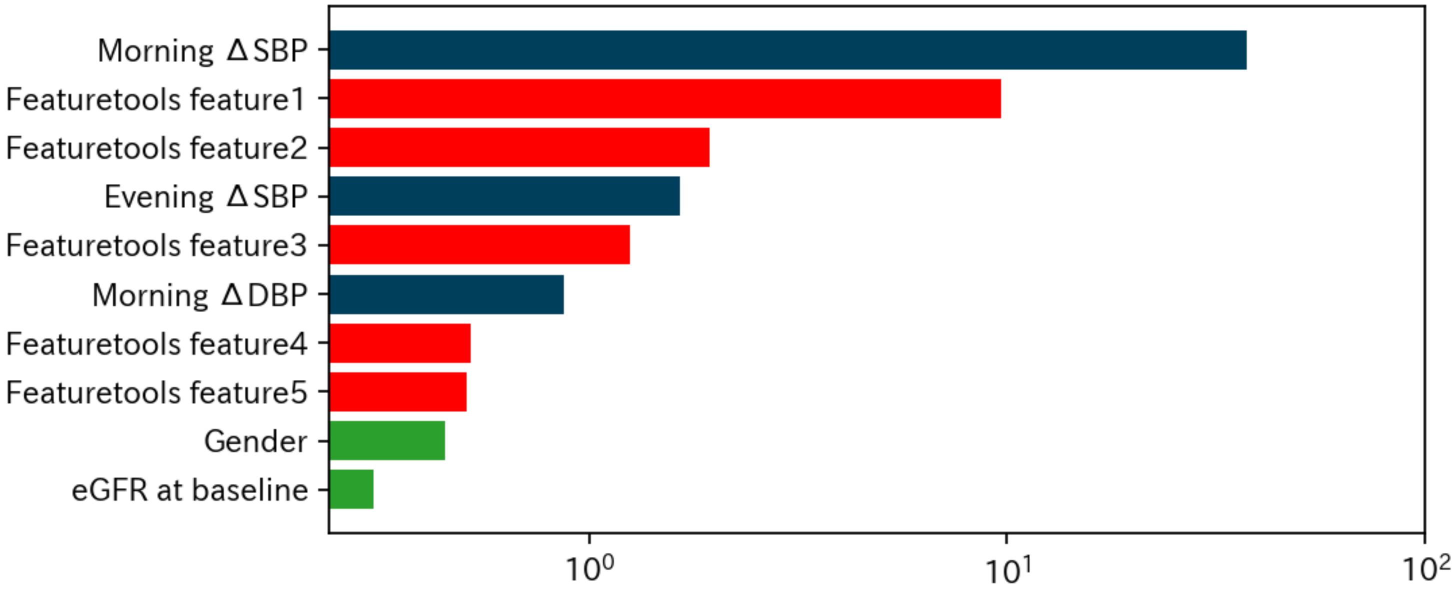

Supplementary Figure 6. Feature importance rankings after feature engineering at 22 weeks.
Bar plot showing the top 10 predictors of systolic blood pressure (SBP) change at 22 weeks after applying feature engineering with feature tools. The red and dark blue bars represent newly generated composite features and original blood pressure-related variables, respectively. Orange bars represent app usage metrics. Green bars represent baseline participant's information. Feature importance is plotted on a log scale because the distribution of importance values is highly right-skewed, and log transformation improves interpretability without altering relative rankings.
Top engineered features include:

- Featuretools feature1 = $(\text{SBP at baseline})^2$ / (Morning SBP)
- Featuretools feature2 = [(SBP at baseline) × (DBP at baseline)] / [(Morning SBP) × (Morning DBP)]
- Featuretools feature3 = (Morning ΔSBP) / (Evening SBP)
- Featuretools feature4 = (Morning ΔSBP) / [(Morning DBP) × $(\text{DBP at baseline})^2$]
- Featuretools feature5 = [(Morning SBP) × (Morning DBP)] / [(SBP at baseline) × (DBP at baseline)]
