# Supplementary material for: Investigating feature-engineered predictors for systolic blood pressure changes in an mHealth-based disease management program
Source: Hypertens Res. 2026 Feb 17;49(4):1204–13. doi: 10.1038/s41440-026-02569-w (PMC13050640; doi:10.1038/s41440-026-02569-w)
Supplement: Supplementary file 2 — Supplementary table [file 41440_2026_2569_MOESM2_ESM.docx]

Supplementary Table 1. Data on app usage metrics throughout the overall program period

|  |  |
| --- | --- |
| Data on app usage |  |
| Session count (sessions/day), n = 2148 | 3.6 (1.7) |
| Time spent per session (min/day), n = 2148 | 1.5 (2.0) |
| Time spent inputting lifelog data (min/day), n = 2127 | 2.6 (3.2) |
| Time spent viewing lifelog data (min/day), n = 2059 | 1.4 (2.8) |
| Time spent viewing home screen (min/day), n = 1132 | 1.3 (2.1) |
| Time spent viewing study materials (min/day), n = 2038 | 0.8 (1.3) |
| Time spent using the chat function (min/day), n = 2117 | 1.6 (1.7) |
| Chat volume (chats/week), n = 2313 | 1.9 (0.6) |
| Meal photo counts (times/week), n = 2225 | 2.1 (1.3) |
| Values are shown as mean (standard deviation). | |

Supplementary Table 2. Maximum correlation with systolic blood pressure change before and after feature engineering.

| Time Point | Before Feature Engineering | After Feature Engineering |
| --- | --- | --- |
|  | r  [95%CI] | r  [95%CI] |
| 4w | 0.455  [0.442, 0.486] | 0.561  [0.533, 0.588] |
| 8w | 0.566  [0.538, 0.593] | 0.602  [0.575, 0.627] |
| 12w | 0.638  [0.613, 0.661] | 0.646  [0.619, 0.673] |
| 22w | 0.849  [0.838, 0.869] | 0.852  [0.839, 0.865] |

Abbreviations: 95%CI, 95% confidence interval.
